# Classification of Traditional Handmade Papers from China, Japan, and Korea Using NIR Hyperspectral Imaging

**DOI:** 10.3390/molecules31111970

**Published:** 2026-06-05

**Authors:** Yong Ju Lee, Seong Bin Park, Seo Young Won, Soon Wan Kweon, Tai-Ju Lee, Hyoung Jin Kim

**Affiliations:** 1 Department of Forest Products and Biotechnology, Kookmin University, 77 Jeongneung-ro, Seoul 02707, Republic of Korea; paperlyj@kookmin.ac.kr (Y.J.L.); dhks615@naver.com (S.W.K.); 2 Intelligent Information Research, Korea High Tech Textile Research Institute, 170, Geomjun-gil, Yangju 11410, Republic of Korea; linus007@koteri.re.kr; 3 Department of Conservation of Cultural Heritage, Kookmin University, 77 Jeongneung-ro, Seoul 02707, Republic of Korea; 203syoung@daum.net

**Keywords:** Hanji, Washi, Xuan paper, NIR hyperspectral imaging, machine learning, SHAP

## Abstract

Traditional handmade papers from China, Japan, and Korea, including Xuan paper, Washi, and Hanji, are difficult to distinguish visually because they share cellulose-rich compositions and similar appearances. This study applied near-infrared hyperspectral imaging (NIR-HSI) and machine-learning classifiers to identify selected traditional handmade papers by country and product type. Spectra in the 1250–1700 nm region were analyzed using k-nearest neighbors, support vector machines, and artificial neural networks. The models achieved high classification performance, with F1-scores of up to 1.000, and Y-scrambling confirmed that the results were not attributable to random class assignment. SHAP analysis identified important wavelength regions near 1256, 1360, 1404, 1449, 1537, 1576, 1635, and 1685 nm, which were associated with C–H, O–H, phenolic, hydrogen-bonded polysaccharide, and lignin-related vibrations. These bands varied among paper groups and provided chemically meaningful information for classification, while SAM visualization revealed pixel-level spectral similarity. These results show that NIR-HSI provides a compact, nondestructive, and interpretable approach for classifying selected East Asian handmade papers.

## 1. Introduction

Handmade paper has long served as a crucial medium for preserving and transmitting human knowledge. Now recognized as an intangible cultural heritage, traditional hand papermaking continues to hold significant cultural, historical, and artistic value [1,2,3]. Distinct traditions have evolved across East Asia, including Chinese Xuan paper [4,5,6], Japanese Washi [7,8], and Korean Hanji [2,9,10], each developed using regionally available plant fibers, tools, and techniques. These practices were typically passed down through families or guilds and gradually adapted to reflect local environments and cultural identities [1,11,12,13,14,15]. The use of diverse plant-based fibers resulted in papers with unique physical and aesthetic properties [2,3,16,17,18]. Although these papers served conventional roles in writing, painting, and calligraphy, their applications have expanded into other domains, including material studies [19,20,21]. However, the accurate identification and classification of traditional papers remain difficult owing to their complex and varied origins. This presents a critical challenge for researchers in cultural heritage, conservation, and forensic science [3,22,23,24].

Handmade papers are primarily composed of cellulose, with varying amounts of residual lignin depending on the fiber source and processing methods. Common raw materials include bamboo, reed hemp, bark, grasses, and paper mulberry [1,24,25]. In Korea, the production of Hanji—a traditional handmade paper—is carried out through a culturally significant and well-preserved process. Typically, one-year-old stems of paper mulberry are steamed and manually peeled to obtain the inner white bark [26]. This bark is then boiled in an alkaline solution prepared from plant-derived ash (e.g., burned soybean, chili, or buckwheat stalks), facilitating the removal of noncellulosic substances such as lignin and pectin. After repeated rinsing, the cleaned fibers are blended with mucilage extracted from *Hibiscus manihot* L. to control the water drainage rate during sheet formation, which is a key factor in producing evenly layered paper. The resulting sheets are conventionally sun-dried on wooden panels [24,27,28]. However, in recent years, cost and supply limitations have driven an increased reliance on imported mulberry and synthetic chemicals such as sodium hydroxide and polyacrylamide [24]. Nevertheless, the core techniques of Hanji production continue to be preserved by skilled artisans and remain an integral part of Korea’s intangible cultural heritage.

Comparable practices also exist in neighboring countries, where regionally adapted techniques have been used to produce handmade papers with similar functional qualities yet distinct cultural identities [1]. The diversity and complexity of traditional papers make it challenging to determine their precise origin or composition via visual inspection alone [3]. Misinterpretations regarding fiber sources date back to the 19th century, when the absence of scientific methodology restricted historical analysis to rudimentary botanical observations. It was not until the early 20th century that material-based investigations were recognized as essential to the study of paper history [3]. This development marked the emergence of an interdisciplinary approach that integrates natural science with cultural heritage studies.

In recent years, scientific characterization techniques have been increasingly applied to handmade papers to better understand their composition and origin. Conventional methods such as optical microscopy [29,30], physical property measurements [2,31], pyrolysis gas chromatography/mass spectrometry [3], elemental analysis [32,33], and size-exclusion chromatography coupled with multiangle light scattering [26], have provided valuable insights into fiber types and quality. However, these methods are inherently destructive, posing risks to fragile or culturally significant samples. To address this limitation, nondestructive approaches have gained prominence, particularly vibrational spectroscopic techniques such as Raman [17,34], near-infrared (NIR) [24], and infrared (IR) spectroscopy [22,35,36]. These methods provide rapid, chemical-specific information without damaging the sample, making them highly suitable for the classification, authentication, and conservation of traditional handmade papers.

Hyperspectral imaging (HSI) is an advanced, noninvasive technique that enables the acquisition of detailed spectral information across numerous contiguous wavelengths [37]. By capturing a complete spectral profile for every pixel [38], HSI allows precise material identification based on their characteristic absorption features [39]. The NIR region, in particular, is highly informative for detecting cellulose and other polysaccharides, which exhibit distinct spectral patterns [39,40,41,42]. This makes HSI a valuable tool for analyzing key structural components in organic materials without the need for direct sampling. The combination of NIR HSI and machine learning (ML) is increasingly being applied in various applications across multiple fields, including food [43], packaging [44], agriculture [45,46], heritage science [23], and materials science [47]. Recently, advances in mathematical algorithms have helped to address challenges in studying organic matter, offering nondestructive alternatives to time-consuming and labor-intensive methods by leveraging diverse spectral information [22,23,24,39,40,41,48,49].

In this study, ML models were constructed based on the NIR spectral data obtained via NIR HSI to classify the manufacturing origin of traditional handmade papers by country and product type. These models were trained to recognize characteristic spectral signatures inherent to the reflectance profiles of the papers, thereby capturing subtle variations associated with different manufacturing practices. The SHapley additive explanations (SHAP) approach was employed to elucidate the internal decision-making process of these models. This method quantified the relative contribution of each wavelength region to the classification outcome, providing a physically interpretable link between spectral information and model response. In addition, the spectral angle mapper (SAM) algorithm was used to visualize the similarity between individual pixel spectra and reference endmembers by evaluating the angular relationships of their spectral vectors. These methods collectively enhance the model transparency and provide interpretative insights into the optical characteristics that underlie the identification of handmade papers.

## 2. Results

### 2.1. Spectral Characteristics of Traditional Handmade Paper

**Figure 1:**
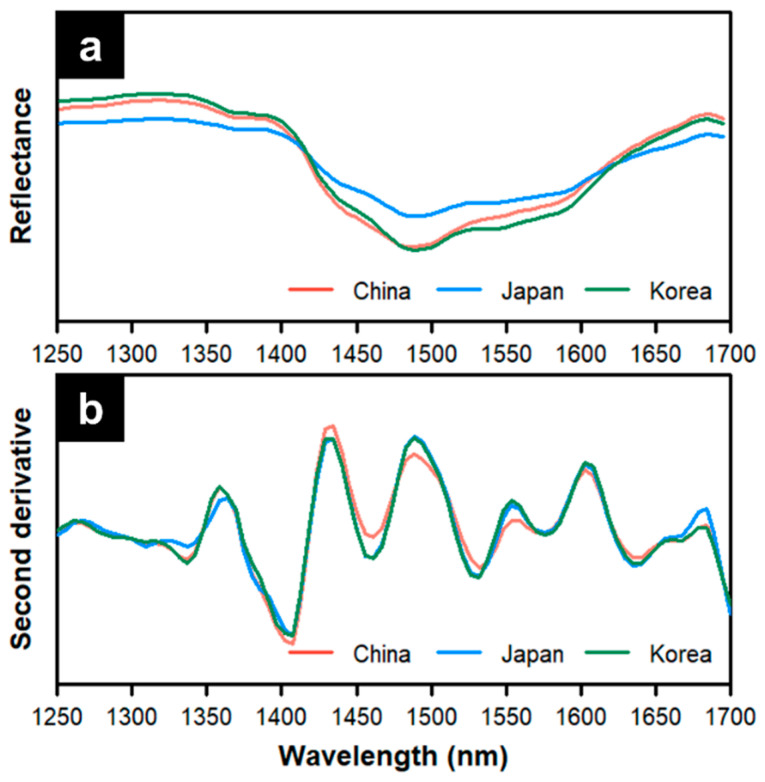
Original (**a**) and second-derivative NIR spectra (**b**) within the 1250–1700 nm region for traditional handmade papers, shown as country-level averages to illustrate representative spectral trends for China, Korea, and Japan.

Figure 1 presents the near-infrared spectra of the traditional handmade papers, shown both as raw reflectance curves and as second-derivative representations (1250–1700 nm). In the original spectra (Figure 1a), the overall shapes are remarkably similar, and the subtle compositional variations originating from differences in fiber sources or manufacturing practices remain largely concealed. This is expected for cellulose-rich materials, where the dominant overtone and combination bands often mask minor structural differences. When the second derivative is applied (Figure 1b), the baseline is effectively stabilized and several latent features emerge with improved clarity, allowing country-specific deviations to be distinguished with greater confidence.

Distinct spectral variations occur around 1390, 1449, 1537, 1576, and 1685 nm. The feature at approximately 1390 nm reflects differences in the amount of physically bound water [50], a parameter strongly governed by fiber morphology and residual hemicellulose content. The broad region between 1420 and 1600 nm corresponds to the first overtone of O–H stretching vibrations [51], and within this interval the band at 1449 nm is associated with phenolic structures [52], consistent with lignin-derived residues characteristic of bast fibers. Peaks at 1537 and 1576 nm arise from hydrogen-bonded polysaccharide domains [24,50,53], reflecting the organization of cellulose microfibrils and the extent of hemicellulose–cellulose interactions. The feature at 1685 nm originates from aromatic ring vibrations in lignin [40], which agrees well with the chemical signatures expected from traditional paper-making fibers.

Although second-derivative preprocessing enhances band resolution and brings chemically meaningful distinctions to the forefront, it also amplifies high-frequency noise and sharpens minor fluctuations. As noted previously, such amplification does not necessarily guarantee improved model performance; in some cases, it may even obscure the relevant variance if not handled with care [54]. This consideration underscores the importance of evaluating machine-learning models on both raw and preprocessed spectra to determine whether derivative enhancement provides genuine analytical benefit.

### 2.2. Principal Component Analysis

**Figure 2:**
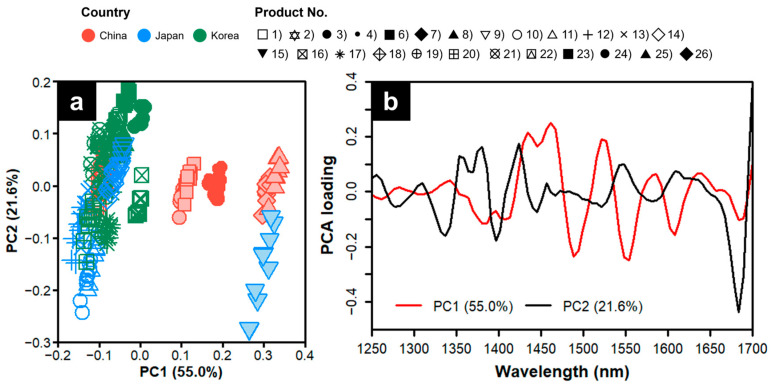
PCA score plot (**a**) of second-derivative NIR spectra and PCA loading plot (**b**) in the 1250–1700 nm region, with class information assigned according to country and product number. The percentages in parentheses on the axis titles represent the explained variance of each PC.

Figure 2a illustrates how the second-derivative NIR spectra of the handmade papers are projected onto the principal-component (PC) space. Traditional handmade papers are produced from a much simpler formulation than modern industrial papers, which typically include a wide range of chemical additives—sizing agents, fillers, retention aids, optical brighteners, and other processing chemicals dispersed within the furnish [55]. In contrast, the fabrication of traditional papers relies almost exclusively on fibrous pulp mixed with modest amounts of natural mucilage [24]. Accordingly, the principal sources of chemical variation among these samples stem from the characteristics of the pulp itself, which are governed by the botanical origin of the fibers and the specific cooking procedures used to separate and purify them [2,22,24,56,57]. The clustered distributions observed in Figure 2a are therefore consistent with similarities in fiber species and cooking agents that determine the relative proportions of cellulose, hemicellulose, and lignin. Common raw materials—such as paper mulberry, bast fibers, bamboo, grasses, and hemp—contribute characteristic chemical signatures that shape these relationships [1,2,24,25].

Figure 2b presents the corresponding PCA loading profiles for the 1250–1700 nm region. A distinct pattern is apparent for the Korean sample (No. 25), which separates from the main cluster along the PC1 axis. This separation is driven by pronounced loadings at approximately 1449 and 1537 nm, wavelengths associated respectively with phenolic structures and hydrogen-bonded polysaccharide domains. Such features indicate differences in pulp chemistry or in the cooking conditions that alter the balance of hemicellulose degradation and cellulose organization. Variations in cellulose crystallinity are particularly sensitive to cooking temperature and alkalinity, which modify the extent and arrangement of hydrogen bonding within the microfibrillar network [58,59]. The band at 1685 nm—attributed to aromatic ring vibrations in lignin—also contributes to sample separation, accounting for the tendency of the Japanese sample (No. 15) to position in the low-PC2 region.

Overall, PCA provides partial separation of the samples, reflecting underlying differences in pulping, bleaching, and fiber composition. However, the predominance of a single unified cluster highlights the limitations of linear dimensionality-reduction methods in capturing the more subtle nonlinearities present in the spectral data. This observation reinforces the need for nonlinear classification models capable of resolving finer-scale chemical and structural distinctions among traditional handmade papers.

### 2.3. Classification Models of Traditional Handmade Paper

To evaluate the classification performance for the traditional handmade papers, three machine-learning approaches—k-NN, SVM, and ANN—were applied. Although SVMs are fundamentally linear classifiers, the use of the RBF kernel [60] enables the model to capture nonlinear relations in the spectral data and thereby provides a more flexible decision boundary.

**Figure 3:**
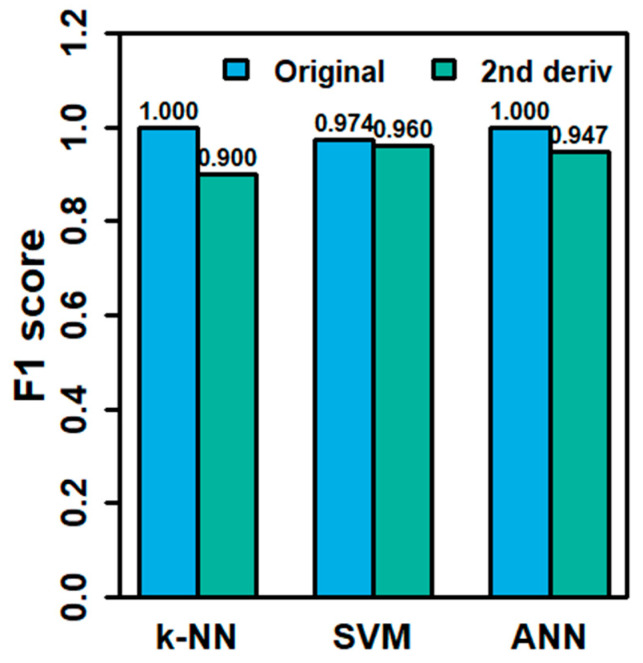
Weighted F1 scores of models for classifying traditional handmade papers. The “2nd deriv” denotes spectra preprocessed using the second-derivative method.

Figure 3 summarizes the F1-score-based comparison of model accuracy for the product-level classification task, and Table 1 compiles the corresponding results for both country and product classification together with the optimal hyperparameters selected for each model. At the manufacturing-country level, all three classifiers achieved perfect discrimination, each yielding an F1 score of 1.000, reflecting the strong chemical separability among the samples when considered at this broader grouping.

More nuanced behavior emerged at the product classification level. For the original spectra, the k-NN and ANN models achieved perfect classification performance, whereas the SVM model showed a slightly lower F1-score. However, when second-derivative preprocessing was applied, the classification performance decreased for all product-level models. This result suggests that the original spectra retained broad reflectance patterns that were useful for distinguishing closely related handmade paper products. Although second-derivative preprocessing can reduce baseline variation and sharpen local absorption features, it may also attenuate broad spectral trends and increase the relative contribution of high-frequency fluctuations. Consequently, the derivative spectra did not consistently improve classification performance in this dataset. This observation is consistent with earlier work showing that derivative preprocessing can amplify noise and obscure relevant spectral variation [54], as also noted in our previous studies [61,62].

While neural networks traditionally attract considerable attention due to their ability to model highly nonlinear relationships, they represent only one possible modeling paradigm [63]. The k-NN classifier, in contrast, is conceptually simple: it assigns labels based on distance relationships in the feature space and does not require iterative parameter training. Neural networks, on the other hand, demand extensive optimization of architectural and training parameters—including the number of layers, neurons, and activation functions—and perform poorly if such tuning is inadequate, often exhibiting sensitivity to overfitting and limited extrapolation capability [64]. Following the principle of Occam’s razor [65], which favors simpler models when predictive performance is comparable, the k-NN algorithm may in practice offer a more parsimonious and equally effective solution for classifying traditional handmade papers compared with the more computationally intensive ANN.

### 2.4. Validation of Classification Models Using Y-Scrambling

**Figure 4:**
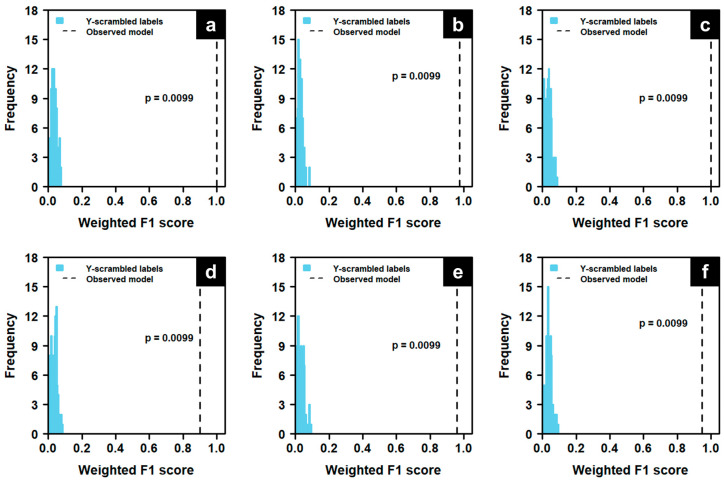
Y-scrambling validation of classification models for traditional handmade paper classification. The distributions show weighted F1-scores obtained from randomly permuted class labels, while the dashed vertical lines indicate the observed model performance. Panels (**a**–**c**) represent the models using the original spectra: (**a**) k-NN, (**b**) SVM, and (**c**) ANN. Panels (**d**–**f**) represent the models using second-derivative spectra: (**d**) k-NN, (**e**) SVM, and (**f**) ANN.

As listed in Table 1, several models employed in this study achieved perfect classification performance, with F1-scores of 1.000. Although these results indicate strong spectral separability among the traditional handmade paper samples, perfect classification performance should be interpreted with caution because it may raise concerns regarding potential overfitting. To address this concern, a Y-scrambling test was additionally conducted (Figure 4).

As shown in Figure 4, the models trained with Y-scrambled labels produced substantially lower weighted F1-scores than the observed models. In all cases, the scrambled-label models showed F1-score distributions close to the lower range, whereas the observed model performances were clearly separated from these null distributions. The permutation-based *p*-values were 0.0099 for all models, indicating that the observed classification performances were unlikely to result from random class assignment. These results suggest that the high F1-scores obtained in the present study were derived from meaningful relationships between the NIR spectral patterns and class labels rather than from simple overfitting.

### 2.5. SHAP-Based Interpretation of Feature Contributions

Although the k-NN classifier produced excellent accuracy with minimal computational demand, its predictions remain essentially descriptive rather than explanatory. Because k-NN assigns labels solely through distance relationships in the feature space, it offers no intrinsic framework for understanding why a particular spectrum is associated with a given paper type. Neural networks are also typically considered black-box models; however, the application of SHAP values enables a more transparent interpretation of their decision pathways [66]. By quantifying the contribution of each input feature—in this case, individual NIR reflectance bands—SHAP highlights the spectral regions most influential for classification.

**Figure 5:**
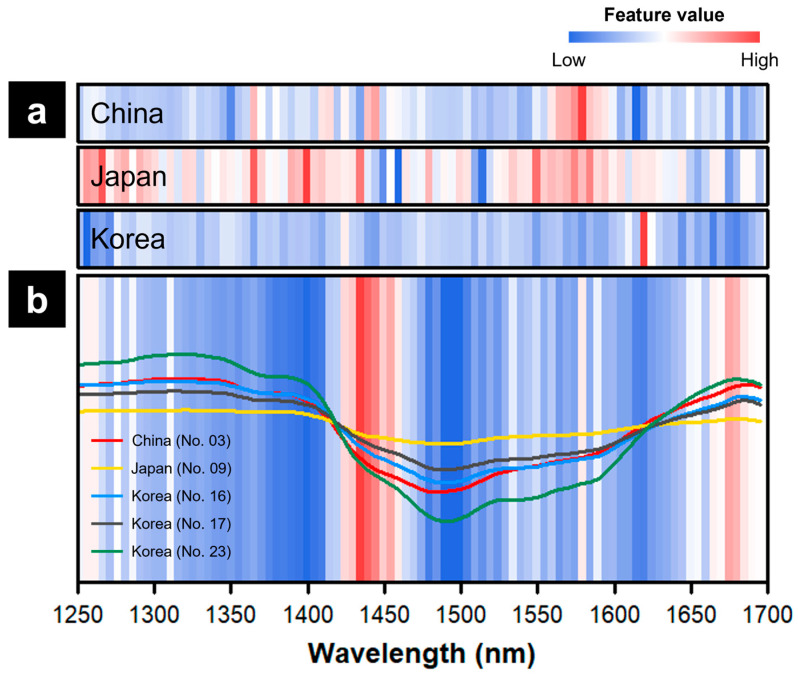
SHAP value heatmaps for the ANN model, where redder colors indicate higher SHAP values: (**a**) country-level classification and (**b**) product-level classification. Representative NIR spectra from five products are also presented.

First, at the country-level classification (Figure 5a), the bands near 1360, 1404, 1449, 1537, and 1576 nm were identified as major contributors to the classification of Chinese and Japanese samples. These bands were associated with C–H stretching and deformation of CH_3_ groups [67], O–H stretching first overtone [68], phenolic structures [52], and hydrogen-bonded polysaccharide domains [24,50,53], respectively. In addition, the bands near 1256 and 1635 nm, corresponding to the second overtone of C–H stretching in CH and CH_2_ groups and lignin-related aromatic and aliphatic C–H vibrations [68], respectively, showed distinct contributions to the classification of Japanese and Korean samples. The SHAP patterns observed for product-type classification (Figure 5b) further revealed specific diagnostic features, including the band near 1685 nm, which is associated with phenolic groups and aromatic ring vibrations in lignin.

Together, these SHAP-derived contributions point toward a coherent chemical interpretation: variations in fiber type, lignin content, polysaccharide composition, and cellulose crystallinity—each governed by differences in cooking intensity, raw-material selection, and subsequent treatments—serve as consistent indicators for distinguishing traditional handmade papers. This convergence of machine-learning interpretability and spectroscopic chemistry provides a clear rationale for the separability observed across samples.

### 2.6. Visualization for Differentiating Traditional Handmade Papers

**Figure 6:**
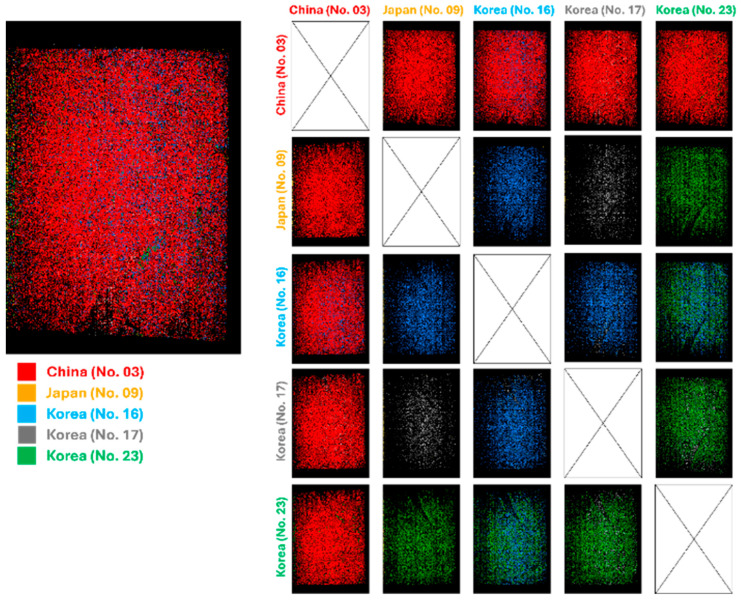
Hyperspectral image-based classification of representative handmade paper samples from China (No. 03), Japan (No. 09), and Korea (Nos. 16, 17, and 23). The composite image on the left illustrates the five-class pixel-wise classification generated using the spectral angle mapper (SAM), with each pixel assigned to the class showing the smallest spectral angle. The matrix on the right presents pairwise binary classifications for each sample combination; white cells denote self-comparisons. These maps highlight spatial variations in spectral similarity and reveal localized chemical distinctions across paper surfaces.

Figure 6 compares the hyperspectral images of representative handmade paper samples, focusing on the same classes used in the feature-importance analysis: China (No. 03), Japan (No. 09), and Korea (Nos. 16, 17, and 23). In these visualizations, each pixel of the hyperspectral cube is color-coded according to the class to which it shows the closest spectral affinity based on the trained classification model.

In the composite five-class map (left panel), the colors appear densely intermixed, reflecting the substantial chemical overlap among the samples when all classes are considered simultaneously. In particular, the Japanese paper showed a relatively weak and less distinct yellow response compared with the Chinese and Korean papers. This behavior reflects the relative nature of SAM, which assigns each pixel to the reference class showing the smallest spectral angle rather than providing an absolute measure of class-specific abundance. Because the Japanese paper shared similar spectral characteristics with some Chinese and Korean papers, its response was partially masked in the merged visualization. Therefore, the unclear yellow response in Figure 6 should not be interpreted as the absence of spectral information, but rather as a limitation of SAM visualization when samples have highly overlapping spectral features. However, when the visualizations are decomposed into pairwise comparisons (right matrix), clearer spatial differentiation emerges. Each panel illustrates the output of minimum-angle matching computed using the SAM algorithm, where colored regions highlight pixels whose spectra exhibit the greatest similarity to a specific reference paper. These visual patterns reveal subtle but meaningful distinctions in fiber composition and local surface chemistry—features that are difficult to perceive from bulk spectral measurements alone.

The contrast between these image-based maps and conventional point-based NIR spectroscopy is instructive. Whereas traditional NIR analysis reduces the sample to a single averaged spectrum, hyperspectral imaging preserves spatial detail, enabling pixel-level scrutiny of heterogeneity within a sheet. This capability provides a powerful means of visualizing chemical similarity, supporting assessments of authenticity, manufacturing consistency, or potential adulteration by mapping how closely unknown regions align with known exemplars.

Taken together, the results highlight the interpretive advantages of the proposed framework. Earlier studies on Hanji classification using IR spectroscopy and PLS-DA required hundreds of spectral variables even after feature reduction [22], and interpretability remained limited. In contrast, the present approach offers both visual transparency and high predictive accuracy. Moreover, when interpretability is not the overriding priority, the inherently simple k-NN classifier continues to provide strong performance at a fraction of the computational cost, emphasizing that model selection should balance complexity with practical considerations.

## 3. Discussion

The results indicate that NIR hyperspectral imaging can distinguish East Asian handmade papers at both broad and fine classification levels, even though the samples share a cellulose-rich composition and visually similar appearance. This outcome is consistent with earlier spectroscopic studies showing that handmade papers retain diagnostically useful chemical information associated with fiber source, cooking intensity, residual lignin, hemicellulose content, and hydrogen-bonding organization [22,24,39,56,57]. The present workflow extends these approaches by combining spectral classification with spatial visualization, allowing both sample-level discrimination and pixel-level inspection of surface heterogeneity. Compared with previous IR-based classification studies, the significance of the present work lies in the integration of compact spectral classification with spatially resolved hyperspectral information. Earlier IR-based Hanji classification studies generally relied on broader wavenumber regions and larger numbers of input variables, such as 425 variables in the 1800–1200 cm^−1^ region for manufacturing-origin classification [69] and 277 variables in the 1800–1500 cm^−1^ region for manufacturer classification [22]. In contrast, the present study achieved high classification performance using only 90 wavelength variables in the 1250–1700 nm NIR region. This dimensional efficiency suggests that the proposed NIR-HSI framework can minimize the computational cost while retaining sufficient chemical information for distinguishing selected traditional handmade papers from China, Japan, and Korea. Moreover, unlike conventional point-based IR spectroscopy, NIR-HSI preserves spatial information, enabling pixel-level visualization of spectral similarity and surface heterogeneity.

A practical advantage of the proposed approach is its nondestructive nature. In museum, conservation, and forensic contexts, sampling is often restricted or impossible; therefore, an imaging-based NIR method can provide rapid screening before more invasive analyses are considered. The use of a simple classifier such as k-NN may be sufficient when the purpose is routine identification of known reference materials, whereas ANN combined with SHAP is more useful when chemical interpretation of the model response is required. The SHAP results suggest that wavelength regions related to O-H, C-H, and lignin-associated vibrations are especially important, supporting the interpretation that classification is driven by meaningful material differences rather than by arbitrary numerical separation. The lower performance observed after second-derivative preprocessing further indicates that preprocessing should be selected according to the spectral characteristics of the dataset rather than applied automatically [54]. In the present case, the original spectra appeared to retain broad reflectance patterns that were useful for classification, whereas derivative preprocessing may have reduced these broad trends while emphasizing local spectral fluctuations. This result suggests that derivative spectra are useful for interpreting local absorption features, but they do not necessarily provide superior predictive performance for NIR-HSI classification of handmade papers.

Several limitations should also be considered. The present study excluded colored papers to minimize interference, and the classification models were developed using a controlled dataset with known sample categories. In addition, the condition of the source lignocellulosic material should be considered. The harvesting stage, drying history, storage condition, and degree of raw-material selection may influence the chemical and structural characteristics of handmade papers. However, in traditional papermaking using paper mulberry, the whole lignocellulosic mass is not generally used directly; rather, the inner bark fibers are separated and processed as the main fiber source. Therefore, the spectral differences observed in this study should be interpreted as reflecting the combined effects of raw-material characteristics and papermaking processes, including fiber selection, cooking, bleaching, and mucilage addition. Pulping conditions have also been reported to affect the crystalline structure and spectroscopic characteristics of cellulose-based fibers [58].

The present study did not include detailed information on the age of the source plants, maceration or fiber-separation conditions, fiber length distribution, soil chemistry, or the chemical composition of the original plant fibers for all samples. These factors may influence the physical and chemical characteristics of handmade papers and may partly contribute to regional or product-level spectral differences. Therefore, the classification results should not be interpreted as being caused solely by geographic origin. Rather, the NIR-HSI models reflect the combined spectral fingerprints of raw-material characteristics, fiber selection, papermaking processes, and preservation conditions in the final paper products.

Historical papers may contain aging products, surface treatments, fillers, pigments, adhesives, conservation materials, inorganic impurities, or moisture gradients that were not represented in the present training set. Although the present manuscript describes the main fiber sources and spectroscopic features related to cellulose, hemicellulose, lignin, bound water, and hydrogen-bonding structures, the quantitative proportion of inorganic impurities in the fibers was not directly measured. Because NIR-HSI primarily reflects organic functional groups and optical responses associated with cellulose-rich paper matrices, it is not sufficient by itself for determining ash content or mineral impurities. Inorganic components may originate from plant-derived ash, soil-derived minerals, fillers, or processing residues, and these factors may influence the optical and chemical characteristics of handmade papers. Because the NIR-HSI measurements in this study were performed in reflectance mode, the obtained spectra should be interpreted as representing the surface and near-surface optical response of the paper rather than the entire fiber volume. The effective penetration depth of NIR radiation can vary depending on wavelength, scattering behavior, paper thickness, density, moisture content, and the presence of surface treatments or additives. Therefore, in historical papers, dyes, pigments, sizing agents, adhesives, conservation materials, degradation products or inorganic components located near the surface may strongly influence the measured spectra. The present study did not quantify the penetration depth of the NIR signal, and this should be considered when extending the proposed model to historical or treated paper artifacts. Because NIR spectra are sensitive to water content, surface condition, and near-surface chemical heterogeneity, future studies should evaluate model robustness under controlled humidity, aging, and treatment conditions. Complementary analyses such as microscopy, compositional measurements, ash content analysis, X-ray fluorescence, inductively coupled plasma analysis, SEM-EDS, cross-sectional imaging, or targeted chemical testing would also help validate whether the spectral differences identified by the models correspond to independent physical or chemical characteristics of the papers.

## 4. Materials and Methods

### 4.1. Traditional Handmade Paper Samples

Table 2 summarizes the traditional handmade paper samples used in this study. Samples containing any form of coloration were excluded to ensure a consistent chemical composition and minimize interference in spectroscopic analysis. Preference was given to the papers obtained from manufacturers who are actively engaged in traditional production practices. Variations in fiber species or production techniques were assumed to influence the chemical and structural characteristics of the papers. Accordingly, the sample selection process was designed to reflect the differences in commonly used plant species and capture variability in key processing steps such as fiber cooking, bleaching, and mucilage preparation methods.

### 4.2. Hyperspectral Image Acquisition and NIR Spectral Dataset

The NIR hyperspectral images (HSI) of each traditional handmade paper were acquired using a Resonon Pika NIR-320 hyperspectral camera (Resonon Inc., Bozeman, MT, USA), covering the spectral range of 900–1700 nm with a spectral resolution of 4.9 nm. Illumination was provided by 120 W halogen light sources. For each paper type, 10 independent paper sheets manufactured from different production lots, manufacturing dates, or manufacturing routes were analyzed. A representative mean reflectance spectrum was extracted from each sheet, resulting in a dataset consisting of 260 NIR spectra from 26 traditional handmade paper types.

Because the regions below 1250 nm were noisy, the spectral range of 1250–1700 nm was used for the classification modeling tasks [40]. The resulting spectra comprised 90 variables corresponding to wavelengths between 1250 and 1700 nm. For the classification modeling, both the original NIR spectra and their second derivatives were used. The second-derivative spectra were obtained using the Savitzky–Golay filter [70], with the derivative order, polynomial order, and smoothing window set to 2, 3, and 7 points, respectively. This preprocessing was applied to enhance local spectral features and suppress baseline effects. Before the classification model construction, L2 normalization was applied to reduce the intensity variations among the spectra.

### 4.3. Principal Component Analysis

Principal component analysis (PCA) was performed to evaluate the differences in the spectral patterns of the traditional handmade papers. PCA transformed the original 90-dimensional NIR spectral data into a set of principal components (PCs), reducing dimensionality while preserving major variance. In this study, five PCs were retained, and each data point was projected onto these new orthogonal axes. Finally, two of these PCs were used to visualize the relationships between the paper types.

### 4.4. Partitioning of NIR Spectral Dataset for Classification Modeling

This study employed two classification levels: country and product. The resulting dataset was structured for country-level classification (3 classes) and product-level classification (26 classes) (Table 2).

The datasets were split into training and test sets at a 7:3 ratio for model development and evaluation, respectively. Stratified random sampling was employed to preserve the class distribution across the sets. Furthermore, a three-fold cross-validation was performed exclusively on the training set to optimize the hyperparameters and assess model stability while preventing overfitting.

### 4.5. Machine Learning Classification Models for Traditional Handmade Paper Using NIR Spectra

**Figure 7:**
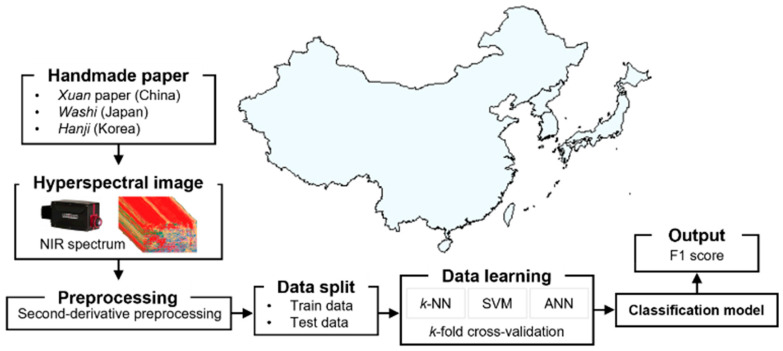
Flowchart of the classification of traditional handmade paper using NIR hyperspectral imaging and machine learning.

To evaluate the classification performance while balancing the computational cost, model transparency, and robustness, various ML algorithms were implemented for the classification of traditional handmade papers. The flowchart of the classification of traditional handmade papers using the combination of HSI and ML is shown in Figure 7.

The k-nearest neighbors (k-NN) algorithm, a distance-based nonparametric method, was employed as a simple and interpretable classifier. The k-NN algorithm assigns labels based on the majority class among the k closest training instances without requiring model training. Odd values of k ranging from 1 to 9 were tested, and the optimal number of neighbors was determined through a grid search.

Support vector machine (SVM) was also used to construct a nonlinear decision boundary by maximizing the margin between classes. A radial basis function (RBF) kernel was applied to map the input data into a higher-dimensional feature space [60]. The hyperparameters for the RBF-SVM, including the penalty parameter (C) and kernel coefficient (gamma), were optimized using grid search across logarithmic scales: C ranging from 100 to 105, and gamma ranging from 10^−1^ to 10^−5^. These hyperparameters control the trade-off between margin maximization and training error and the influence of individual data points in the kernel function.

A multilayer feedforward artificial neural network (ANN) with backpropagation was also adopted. The rectified linear unit function was used for activation, and cross-entropy was selected as the loss function. The model optimization was performed using the adaptive moment estimation optimizer. Various network configurations with one or two hidden layers and differing numbers of neurons were evaluated to identify the best-performing architecture. The initial learning rates of 0.0001, 0.001, 0.01, and 0.1 were evaluated, and the maximum number of training iterations was set to 300.

For visualization purposes, the SAM algorithm [71] was applied. The SAM calculates the spectral similarity between each pixel and the reference spectra (endmembers) by measuring the angle between their spectral vectors. This process produces one raster layer per endmember, where each pixel value represents the spectral angle. A smaller angle indicates higher similarity between the pixel and the corresponding endmember class. In the subsequent step, a classification map was generated by assigning each pixel to the endmember with the smallest spectral angle, a process referred to as minimum-angle classification. Optionally, the classification performance can also be refined by applying a maximum angle threshold to exclude pixels with low spectral similarity.

### 4.6. SHAP-Based Model Explainability Analysis

To interpret the influence of each wavelength variable on the classification decision, the SHAP algorithm was employed [66]. SHAP builds upon cooperative game theory by assigning a Shapley value to each feature, representing its marginal contribution to the overall prediction. This additive framework enables the model-agnostic interpretability of complex ML models. In this study, SHAP analysis was applied to the optimized NIR classification model to visualize the importance and directionality of the wavelength features used to classify traditional handmade papers.

### 4.7. Evaluation Metrics for Classification Models

In binary classification, prediction outcomes are typically categorized based on their correctness. A correctly predicted instance from the positive class is referred to as a true positive (TP), and an accurately classified negative instance is known as a true negative (TN). A positive instance mistakenly predicted as negative is termed a false negative (FN), and a negative instance incorrectly predicted as positive is referred to as a false positive (FP) [72].

F1 score is commonly employed to evaluate the performance of classification models, particularly in the context of imbalanced datasets [73]. Unlike overall accuracy, which can be misleading in the presence of class imbalance due to the dominance of majority classes, the F1-score provides a more balanced assessment. F1-score is defined as the harmonic mean of precision and recall, which is calculated as follows:

Precision = TP/(TP + FP),(1)

Recall = TP/(TP + FN),(2)

F1-score = 2 × (Precision × Recall)/(Precision + Recall),(3)

w*_i_* = n*_i_*/N, (4)

Weighted F1-score = ∑*_i_* w*_i_*F1*_i_*. (5)

where w*_i_* denotes the weight of class *i*, n*_i_*, denotes the number of samples in class *i*, N denotes the total number of samples, and F1*_i_* denotes the class-specific F1-score.

All data processing and classification modeling tasks were performed using the R statistical software (R Core Team, ver. 4.4.2, Auckland, New Zealand).

### 4.8. Y-Scrambling Test for Model Validation

A Y-scrambling test was conducted to assess whether the high classification performance resulted from overfitting or random class assignment. The class labels were randomly permuted while the NIR spectral variables were kept unchanged. For each scrambled dataset, the same preprocessing, training/test partitioning, cross-validation, and hyperparameter optimization procedures used for the original models were applied. This process was repeated 100 times for each model and preprocessing condition to generate a null distribution of weighted F1-scores. The observed weighted F1-scores were then compared with the scrambled-label distributions, and permutation-based *p*-values were calculated to evaluate whether the observed performances were significantly higher than those expected by chance.

## 5. Conclusions

This study demonstrated that NIR-HSI combined with machine learning can nondestructively classify selected traditional handmade papers from China, Japan, and Korea. Using 90 wavelength variables in the 1250–1700 nm region, the models achieved high classification performance, and Y-scrambling supported that the results were not caused by random class assignment. SHAP highlighted diagnostic bands near 1256, 1360, 1404, 1449, 1537, 1576, 1635, and 1685 nm, indicating that classification was associated with C–H, O–H, phenolic, polysaccharide, and lignin-related spectral features. SAM further enabled pixel-level visualization of spectral similarity and surface heterogeneity. Although broader validation using historical, treated, and compositionally diverse papers is still needed, the proposed framework offers a compact and noninvasive strategy for traditional paper classification.

## Figures and Tables

**Table 1:** Performance comparison of classification models (k-NN, SVM, and ANN) for traditional handmade paper, including optimal hyperparameter combinations.

CL	Preproc.	Model	F1 Score	Hyperparameters
Country	Original	k-NN	1.000	k = 5
Country	Original	SVM	1.000	C = 10^4^, gamma = 10^−3^
Country	Original	ANN	1.000	hl_size = (16), lr = 0.01
Country	Second derivative	k-NN	1.000	k = 3
Country	Second derivative	SVM	1.000	C = 10^1^, gamma = 10^−2^
Country	Second derivative	ANN	1.000	hl_size = (16), lr = 0.001
Product	Original	k-NN	1.000	k = 1
Product	Original	SVM	0.974	C = 10^3^, gamma = 10^−1^
Product	Original	ANN	1.000	hl_size = (16), lr = 0.1
Product	Second derivative	k-NN	0.900	k = 5
Product	Second derivative	SVM	0.960	C = 10^2^, gamma = 10^−2^
Product	Second derivative	ANN	0.947	hl_size = (32), lr = 0.01

Notes: CL, classification level; Preproc., preprocessing; C, cost; hl_size, hidden layer size; lr, learning rate.

**Table 2:** List of traditional handmade paper samples analyzed.

Code.	Country	Product Name	Pulp Fiber
China (No. 01)	China	Dakji	paper mulberry
China (No. 02)	China	Dakji	paper mulberry
China (No. 03)	China	Sangpiji	paper mulberry
China (No. 04)	China	Sangpiji	paper mulberry
China (No. 05)	China	Myeonryoji	paper mulberry
China (No. 06)	China	Myeonryoji	paper mulberry
China (No. 07)	China	Jukji	bamboo
China (No. 08)	China	Jukji	bamboo
Japan (No. 09)	Japan	Sekishu paper	paper mulberry
Japan (No. 10)	Japan	Mino-washi	paper mulberry
Japan (No. 11)	Japan	Mino-washi	paper mulberry
Japan (No. 12)	Japan	Misu-washi	paper mulberry
Japan (No. 13)	Japan	Misu-washi	paper mulberry
Japan (No. 14)	Japan	Uda paper	paper mulberry
Japan (No. 15)	Japan	Uda paper	paper mulberry
Korea (No. 16)	Korea	Pulp hanji	paper mulberry, wood pulp
Korea (No. 17)	Korea	Hanji	paper mulberry
Korea (No. 18)	Korea	Olbal Hanji	paper mulberry
Korea (No. 19)	Korea	Olbal Hanji	paper mulberry
Korea (No. 20)	Korea	Ssangbal Hanji	paper mulberry
Korea (No. 21)	Korea	Hanji—Choksae	paper mulberry
Korea (No. 22)	Korea	Hanji—Choksae	paper mulberry
Korea (No. 23)	Korea	Eumyungji	paper mulberry
Korea (No. 24)	Korea	Eumyungji	paper mulberry
Korea (No. 25)	Korea	Eumyungji	paper mulberry
Korea (No. 26)	Korea	Eumyungji	paper mulberry

## Data Availability

The datasets and codes generated and analyzed during the current study are publicly available in the GitHub repository [74]. The analyses were performed using R version 4.4.2.
